# A Multi-Feature Fusion Approach for Road Surface Recognition Leveraging Millimeter-Wave Radar

**DOI:** 10.3390/s25123802

**Published:** 2025-06-18

**Authors:** Zhimin Qiu, Jinju Shao, Dong Guo, Xuehao Yin, Zhipeng Zhai, Zhibing Duan, Yi Xu

**Affiliations:** 1 School of Transportation and Vehicle Engineering, Shandong University of Technology, Zibo 255049, China; qiuzm1123@163.com (Z.Q.); guodong@sdut.edu.cn (D.G.); 15963252846@163.com (X.Y.); zhipengzhai31@163.com (Z.Z.); zhibingduan6@gmail.com (Z.D.); xuyi@sdut.edu.cn (Y.X.); 2 The Intelligent Connected Vehicle Laboratory, Shandong University of Technology, Zibo 255049, China

**Keywords:** millimeter-wave radar, road surface recognition, wavelet transform, statistical features, machine learning

## Abstract

With the rapid progress of intelligent vehicle technology, the accurate recognition of road surface types and conditions has emerged as a crucial technology for improving the safety and comfort levels in autonomous driving. This paper puts forward a multi-feature fusion approach for road surface identification. Relying on a 24 GHz millimeter-wave radar, statistical features are combined with wavelet transform techniques. This combination enables the efficient classification of diverse road surface types and conditions. Firstly, the discriminability of radar echo signals corresponding to different road surface types is verified via statistical analysis. During this process, six-dimensional statistical features that display remarkable differences are extracted. Subsequently, a novel radar data reconstruction approach is presented. This method involves fitting discrete echo signals into coordinate curves. Then, discrete wavelet transform is utilized to extract both low-frequency and high-frequency features, thereby strengthening the spatio-temporal correlation of the signals. The low-frequency information serves to capture general characteristics, whereas the high-frequency information reflects detailed features. The statistical features and wavelet transform features are fused at the feature level, culminating in the formation of a 56-dimensional feature vector. Four machine learning models, namely the Wide Neural Network (WNN), K-Nearest Neighbors (KNN), Support Vector Machine (SVM), and Kernel methods, are employed as classifiers for both training and testing purposes. Experiments were executed with 8865 samples obtained from a real-vehicle platform. These samples comprehensively represented 12 typical road surface types and conditions. The experimental outcomes clearly indicate that the proposed method is capable of attaining a road surface type identification accuracy as high as 94.2%. As a result, it furnishes an efficient and cost-efficient road perception solution for intelligent driving systems. This research validates the potential application of millimeter-wave radar in intricate road environments and offers both theoretical underpinning and practical support for the advancement of autonomous driving technology.

## 1. Introduction

### 1.1. Motivation

Accurate identification of road surface types can provide reliable decision-making support for intelligent driving, significantly enhancing driving safety and stability while optimizing driving efficiency and ride comfort. This capability strongly promotes the innovation and development of intelligent vehicle technology [1].

Currently, methods for identifying road surface types can be mainly classified into three categories: direct identification, indirect identification, and multi-source information fusion.

Direct identification methods depend on sensors like vision sensors, lidar, and ultrasonic radar to directly sense road surface information and then classify road types accordingly. However, due to inherent limitations in sensor hardware, vision sensors often exhibit reduced robustness under adverse environmental conditions, such as varying lighting, weather impacts, or debris interference [2]. Lidar systems involve substantial hardware costs, generate massive volumes of data that require intensive processing, and necessitate high-performance computing units to support real-time analysis [3]. Ultrasonic radar is inherently limited by its wide scattering angle and low directivity.

Indirect road surface identification primarily relies on analyzing in-vehicle dynamic parameters during travel—such as speed, acceleration, and sideslip—to infer road surface types. However, a critical limitation of indirect methods is their reliance on real-time vehicle–surface interaction data, which restricts them to characterizing the current road condition without predictive capability for upcoming segments [4].

Multi-sensor fusion classification methods, which rely on the information interaction inherent in multi-sensor frameworks, further undermine the real-time decision-making efficiency of vehicles [5]. This is because the complex data integration typically requires substantial computational resources and incurs latency.

As evident from the above, lighting and weather interference, sensor limitations, and insufficient sample diversity are the key factors limiting road surface recognition. Existing road identification methods generally only recognize a limited number of common road surfaces and are ineffective in detecting road moisture states.

**Figure 1:**
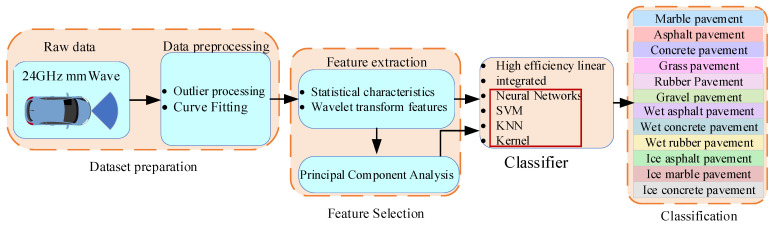
Identification model flow chart.

This paper proposes a new low-cost method for road surface identification, which leverages 24 GHz millimeter-wave radar signals by fusing statistical features and wavelet transform techniques to achieve accurate classification of both road types and their moisture states, as depicted in Figure 1.

### 1.2. Literature Review

Direct identification methods depend on sensors like vision sensors, lidar, and ultrasonic radar to directly sense road surface information and then classify road types accordingly. Vision sensor-based road surface type identification generally involves extracting texture features from road images, followed by training classification models using traditional machine learning techniques such as K-Nearest Neighbors (KNN) and Support Vector Machines (SVM) [6,7]. With the rise of deep learning, Convolutional Neural Network (CNN)-based approaches have increasingly replaced conventional machine learning methods, providing superior detection accuracy and real-time performance capabilities [8,9,10].

In specific research, Liu and Huang collected wet road surface image samples and developed an SVM-based classification model for slippery road condition detection [11]. Their model showed a higher misclassification rate under dry surface conditions but achieved significantly better accuracy for snow-covered surfaces. Wan et al. proposed a road slip state discrimination model using a Radial Basis Function (RBF) neural network, which demonstrated moderate classification accuracy for dry and mud-covered surfaces but notably higher precision for icy and snowy road conditions [12]. Wang et al. utilized vision sensors to establish a dataset and introduced a road classification model based on structural reparameterization and adaptive attention mechanisms, enabling fast and accurate identification of complex road surfaces ahead of the vehicle [13].

Lidar technology, renowned for its high detection accuracy and robust resilience to environmental variations, has gained widespread adoption in road surface identification tasks [14]. The intensity of lidar echo signals demonstrates strong discriminative power due to inherent differences in road surface materials, hardness, and smoothness, enabling effective classification of diverse road conditions [15]. Lalonde et al. pioneered the application of lidar sensors in road surface type classification by developing a terrain classification method for vegetated environments using 3D point cloud data, marking an important milestone in this research domain [16]. However, the drawbacks of lidar systems have, to some extent, hindered their large-scale implementation in cost-sensitive or resource-restricted scenarios. Ultrasonic radar, akin to millimeter-wave radar, employs echo signal analysis to distinguish road surface types [17,18]. The surface roughness and acoustic impedance of different road materials directly influence the propagation and reflection characteristics of ultrasonic waves, enabling the extraction of material-specific features through signal processing [19]. Specifically, variations in these physical properties create distinguishable patterns in the reflected signals, which serve as the basis for classification. However, ultrasonic radar diminishes measurement accuracy when identifying road surfaces beyond short ranges, as the broad beam spreads energy diffusely and reduces the precision of signal interpretation.

Indirect road surface identification primarily relies on analyzing in-vehicle dynamic parameters during travel—such as speed, acceleration, and sideslip—to infer road surface types [20,21,22]. For example, Ao et al. leveraged onboard gyroscopes and speed sensors to collect motion data including roll and pitch angles and then employed a Backpropagation (BP) neural network to classify road types based on these kinematic features [23]. Wang et al. defined six characteristic intervals representing typical road surface properties and developed a 14-degree-of-freedom vehicle dynamics model incorporating road roughness excitation, enabling effective real-time classification of surface conditions. In acoustic-based approaches [24]. Gailius et al. systematically collected tire–road friction noise samples under controlled environments, establishing a physical correlation between noise characteristics and road friction coefficients through spectral energy distribution and dominant frequency analysis [25]. Similarly, Alonso et al. captured tire acoustic signals using sensors, extracted features via statistical methods and the Mel-Frequency Cepstral Coefficient (MFCC) algorithm, and trained Support Vector Machine (SVM) and Artificial Neural Network (ANN) classifiers to identify road surfaces based on noise patterns [26].

However, approaches based on vehicle dynamics or acoustic measurements share this fundamental constraint—they provide no foresight into road surfaces ahead, limiting their utility in proactive safety systems that require pre-emptive information about changing road conditions.

Each road surface classification method—whether relying on vehicle dynamics response, vision sensors, lidar, or ultrasonic radar—possesses unique advantages. To this end, researchers have sought to integrate multi-source information into classification frameworks, aiming to enhance the accuracy of traversability prediction by leveraging the complementary strengths of diverse sensing modalities. Zhang Lei et al. integrated a tire-model-based road adhesion coefficient estimator with an image recognition-based forward adhesion coefficient prediction method, achieving road type identification via a convolutional neural network [27]. Safont G et al. utilized four distinct sensor modalities, individually extracting features from each before combining them into a unified dataset, followed by a feature selection process prior to classification [28]. However, the heightened complexity of information interaction among heterogeneous data sources complicates the classification algorithm architecture, thereby compromising the vehicle’s real-time control decision-making efficiency.

However, multi-sensor fusion classification methods are generally governed by the barrel principle, where the performance ceiling is dictated by the weakest sensor or processing component. Additionally, the increased complexity of information interaction inherent in multi-sensor frameworks further impairs the real-time decision-making efficiency of vehicles, as intricate data integration often demands more computational resources and introduces latency.

Existing road identification methods generally only recognize a limited number of common road surfaces and are ineffective in detecting road moisture states. This paper introduces a novel feature extraction approach for road identification, which leverages 24 GHz millimeter-wave radar signals by fusing statistical features and wavelet transform techniques to achieve accurate classification of both road types and their moisture states.

### 1.3. Contributions

The core contributions of this work are as follows:

1. This study explores the feasibility of employing a low-cost, high-efficiency 24 GHz millimeter-wave radar for road surface type and state identification. Analysis of radar echo signals reveals pronounced disparities in echo intensity among different road types (e.g., asphalt, gravel) and moisture states (dry, wet, ice). By extracting statistical features—such as mean, variance—from these signals, strong class separability is achieved across various road conditions. Notably, the use of a 24 GHz millimeter-wave radar to identify road moisture states has been rarely explored in prior research, representing a key innovation of this work.
2. This study reconstructs radar data as time-series sequences, converting discrete radar signals into curve-based image representations that visualize signal dynamics over time. Wavelet transform is then applied to these resulting curve images to extract multi-scale features, effectively capturing both low-frequency trends (e.g., long-term signal patterns) and high-frequency details (e.g., transient reflections). This approach significantly enhances the temporal–spatial correlation of echo information, reducing the sensitivity to localized road surface irregularities or damage and providing a robust, novel framework for road surface recognition.
3. Feature-level fusion is achieved by integrating wavelet transform-derived multi-scale features with statistical characteristics extracted from raw radar signals, generating richer and more discriminative feature vectors for road condition analysis. This integrated strategy significantly enhances the accuracy and robustness of road surface identification by leveraging the complementary information from both time-domain statistics and frequency-domain structural details.

### 1.4. Paper Organization

The second part of the paper conducts separability analysis on the millimeter-wave radar echo intensity signal and extracts features based on statistics. The third part of the paper extracts features based on wavelet transform. The fourth part of the paper performs feature-level fusion and constructs a machine learning classifier. In the fifth section, the model undergoes verification through real-world vehicle testing. Finally, the study culminates with conclusive remarks, encapsulating the research findings and implications.

## 2. Feature Extraction Based on Statistics

### 2.1. Road Surface Separability Analysis of 24 GHz Millimeter-Wave Radar

Considering the substantial disparities in materials employed for diverse road surfaces, the hardness characteristics, dielectric constants, and surface roughness of these surfaces manifest unique properties. Consequently, the strength of the echo signals detected by millimeter-wave radar on different road surfaces is expected to exhibit considerable variations.

In order to verify the recognition ability of 24 GHz millimeter-wave radar under different road surfaces and conditions, this study conducted experiments on various road surface types. The experimental site is a fixed and enclosed location on campus, with sunny days; a wind speed of <3 m/s; the radar installed at the center of the front cover, 15 cm from the ground; a pitch angle of 45°; the radar refresh rate set to 20 Hz; and 64 sampling points. During data collection, the vehicle traveled at a constant speed of 15 m/s, and GPS/INS synchronously triggered data recording.

**Figure 2:**
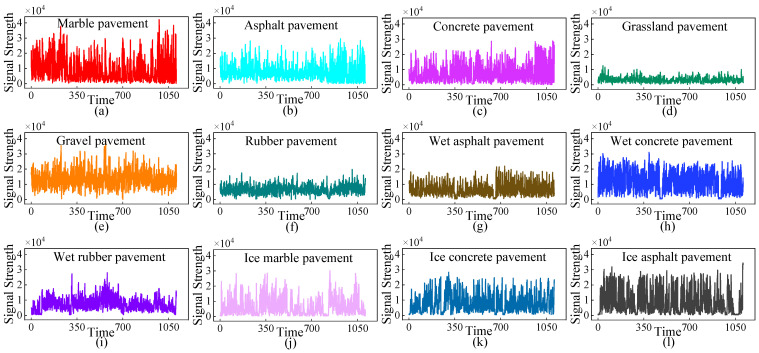
Echo signal diagram of different road types.

The experimental roads include marble, rubber, asphalt, concrete, gravel, grass, wet rubber, wet asphalt, wet concrete, ice marble pavement, ice asphalt pavement, and ice concrete. Due to the low installation position of the radar and the dense ground reflection, the received signal power would increase significantly locally. Through in-depth analysis of the road echo signal distribution shown in Figure 2, it can be found that the intensity corresponding to the radar echo signal under different road surfaces and conditions is significantly different, which fully demonstrates the excellent resolution ability of the radar.

The echo strength of the grass surface exhibits a relatively concentrated distribution, with the lowest signal strength values predominantly falling within the 0–10,000 dB range. This suggests that the grass surface features excellent continuity and regularity. For asphalt and rubber surfaces, the echo strength generally remains below 25,000 dB and shows a more dispersed pattern. This dispersion can be attributed to the multitude of cracks present on these surfaces.

In contrast, marble, concrete, and gravel surfaces display relatively higher echo strengths. These surfaces, characterized by their hardness and low friction coefficients, cause minimal scattering of radar signals. Nevertheless, the distribution of their echo strengths is also rather scattered, mainly because of surface cracking, aging, and wear over time.

Wet road surfaces, owing to the elevated water content, possess a relatively high dielectric constant. This property leads to increased absorption and scattering of radar signals, consequently impacting the strength of the radar echoes.

Frozen road surfaces typically feature enhanced surface roughness and irregularities, which can significantly affect the reflection of radar signals. The ice surface not only has the potential to partially absorb radar signals but also causes the scattering of these signals. This dual effect of absorption and scattering on frozen road surfaces ultimately results in a reduction in the strength of radar return signals.

Through comprehensive analysis of millimeter-wave radar echo signal data and the application of sophisticated model algorithms, it is possible to achieve highly accurate detection and in-depth classification of various road surfaces.

### 2.2. Statistical Feature Extraction

**Figure 3:**
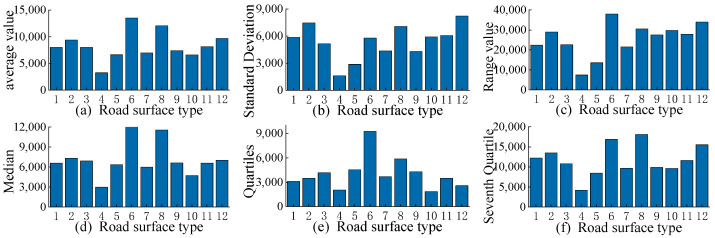
Comparison of Echo Signal Statistical Features.

The basis of road surface recognition lies in the extraction of road features. This paper uses statistical analysis methods to extract radar echo signal features of different road types. Based on prior knowledge, six features are determined: mean, standard deviation, range, median, quartile, and septile. The extracted feature information is shown in Figure 3 and Table 1. The following is the calculation formula for each feature index.

Assume that the radar echo signal amplitude sequence is ${\left\{x_{i}\right\}}_{i=1}^{N}$; among them, $x_{i}$ represents the *i* th sampling value; *N* represents the total number of samples.

1. Mean (μ): The arithmetic mean of the sample signal strength, calculated as follows:

(1) $$\tilde{x}=\frac{1}{N}\sum_{i=1}^{N}x_{i}$$

2. Standard Deviation (σ): An unbiased estimate of the sample signal strength, calculated as follows:

(2) $$\sigma =\sqrt{\sum_{i=1}^{N}\left(x_{i}-\mu \right)/N-1}$$

Among them, μ is the sample mean, and the denominator is N−1 to eliminate estimation bias.

3. Range: The difference between the maximum and minimum values, calculated as follows:

(3) $$Range=\underset{1\leq i\leq N}{\max x_{i}}-\underset{1\leq i\leq N}{\min x_{i}}$$

Among them, $\max x_{i}$ and $\min x_{i}$ are the maximum and minimum values of the sequence, respectively.

4. Median: Arrange all N echo amplitude values in ascending order.

If N is an odd number, the median is the number in the middle after arrangement; if N is an even number, the median is the average of the two middle numbers after arrangement.

5. Quartiles: Arrange all the values in the sample from small to large and divide them into four equal parts. After all the values in the sample are arranged from small to large, the number that is around 75% is found.
6. Seventh Quartile: Arrange all the values in the sample from small to large and divide them into four equal parts. After all the values in the sample are arranged from small to large, the number that is around 75% is found.

## 3. Feature Extraction Utilizing Wavelet Transform

### 3.1. Curve Fitting Graphical Modeling

Wavelet transform has the capability to decompose signals or images into frequency components across various scales. This unique property endows it with substantial advantages in the realm of image processing, especially in tasks related to feature extraction and classification, as evidenced by previous studies.

Radar echo signals are essentially discrete data. Discrete signal strength signals cannot usually be used directly for road classification. Moreover, the original discrete radar signals may have certain noise and intermittent distortion. Wavelet transform has more significant advantages in processing continuous signal data. In order to apply wavelet transform to road type recognition, the characteristic curve information of different road types can be extracted by sampling these discrete data at uniform time intervals and fitting them into quadratic curves. The characteristic curves of the same road type show obvious similarities, while the characteristic curves of different road types show different low-frequency and high-frequency patterns over time.

Applying wavelet transforms to the generated curve images for feature extraction enables the separation and acquisition of low-frequency and high-frequency information. This process facilitates the extraction of abundant features that are not discernible in the original discrete radar signals, effectively enhancing the temporal and spatial correlations within the echo information. As a result, this approach can mitigate the adverse effects of unexpected road damage on road recognition outcomes, thereby presenting a novel and effective method for pavement recognition.

Furthermore, wavelet transform proves effective in extracting the unique low-frequency and high-frequency information revealed by the echo signal curves of different road surfaces over time. The low-frequency component is utilized as feature information, whereas the high-frequency component acts as detailed information.

**Figure 4:**
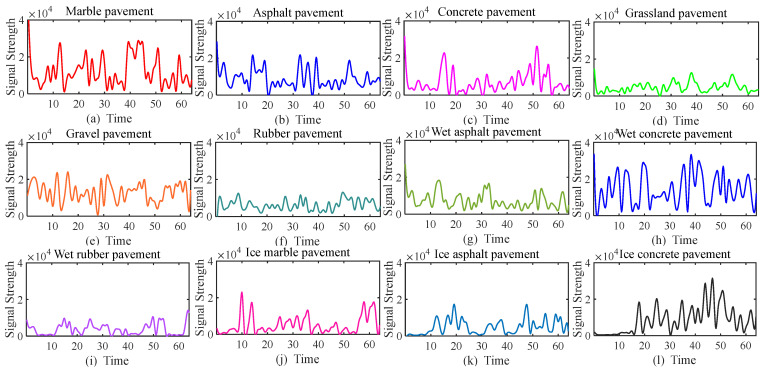
Fitting diagrams of signals on different road surfaces.

For road surface recognition, the echo signal contains 64 sampling moments, including the current moment and 63 historical sampling moments. Then, the 1 × 64 sampling data is processed. A 1 × 8 sampling window is used to collect road surface perception data in a rolling manner. Each time the window moves, eight new sampling data points are added to the fixed matrix, and the last eight echo data points in the matrix are removed. This achieves 87.5% window overlap (i.e., 56 data points are retained). This strategy ensures the continuity of timing information and the stability of model output [29,30]. Considering the spatial continuity of the road surface, quadratic curve fitting is used to convert the discrete echo matrix into a continuous curve representation, as shown in Figure 4.

### 3.2. Feature Extraction Based on Wavelet Transform

Wavelet transform is a powerful signal analysis technique renowned for its time–frequency locality and multi-resolution analysis capabilities [31,32]. It offers superior time–frequency positioning, enabling it to unveil the intricate local features of signals. While continuous wavelet transform (CWT) has the advantage of exploring all possible scale and translation parameters, it demands substantial computational resources and presents challenges in practical processing.

In this study, discrete wavelet transform (DWT) is adopted as a more viable alternative. DWT significantly reduces computational complexity and facilitates multi-resolution analysis through binary scaling and translation operations [33,34]. This method provides excellent time resolution at high frequencies, allowing for precise temporal signal analysis, and offers good frequency resolution at low frequencies, enabling detailed spectral characterization.

This paper applies the feature extraction technology of discrete wavelet transform to the curves that characterize the road surface information. DWT uses a single function called the mother function to decompose the signals of different road surface types into several functions as shown in the expression [35,36,37,38]:

(4) $$\psi \left(t\right)=\frac{1}{\sqrt{2}}\psi \left(\frac{t-y}{z}\right)x,y\in S,x\geq 0$$

where *x* and *y* are the scaling and shifting parameters, respectively, and *S* is the wavelet space. The wavelet transform is shown in the following equation [39,40]:

(5) $$F\left(x,y\right)=\frac{1}{\sqrt{x}}\int \psi \left(\frac{t-y}{x}\right)dt$$

When the number of wavelet transform layers is set to 2, the original signal is first decomposed by applying the low-pass and high-pass filters of the Daubechies 1 wavelet. Daubechies wavelet: The corresponding analysis and synthesis filters contain only two sets of coefficients, with the shortest support interval, covering only one sampling interval. In addition, the Db1 transform only requires addition and subtraction operations, with the lowest amount of calculation. This is of great significance to real-time systems. Since the Db1 wavelet filter spans only two points, it has a strong suppression effect on high-frequency noise (internal and environmental stray noise of the radar system). This step decomposes the signal into wavelet coefficients of different scales. By recursively applying low-pass and high-pass filters to the signal followed by downsampling (i.e., sampling every other data point), the algorithm obtains the approximate coefficient A1 (representing the low-frequency component) and the detail coefficient D1 (corresponding to the high-frequency component) of the signal [41].

(6) $$F\left(t\right)=\sum_{k=-\infty }^{k=+\infty }D_{n,k}\phi \left(2^{-n}t-k\right)+\sum_{k=-\infty }^{k=+\infty }\sum_{k=-\infty }^{k=+\infty }2^{\frac{-j}{2}}A_{j,k}\psi \left(2^{-j}t-k\right)$$

The algorithm then continues with a second-level decomposition of the approximation coefficients. This is achieved by another round of filtering and downsampling using the same set of filters. This results in higher-level approximation coefficients and detail coefficients, and the algorithm is iterated in this process to achieve multiple levels of decomposition, leading to a deeper analysis of the road surface information.

**Figure 5:**
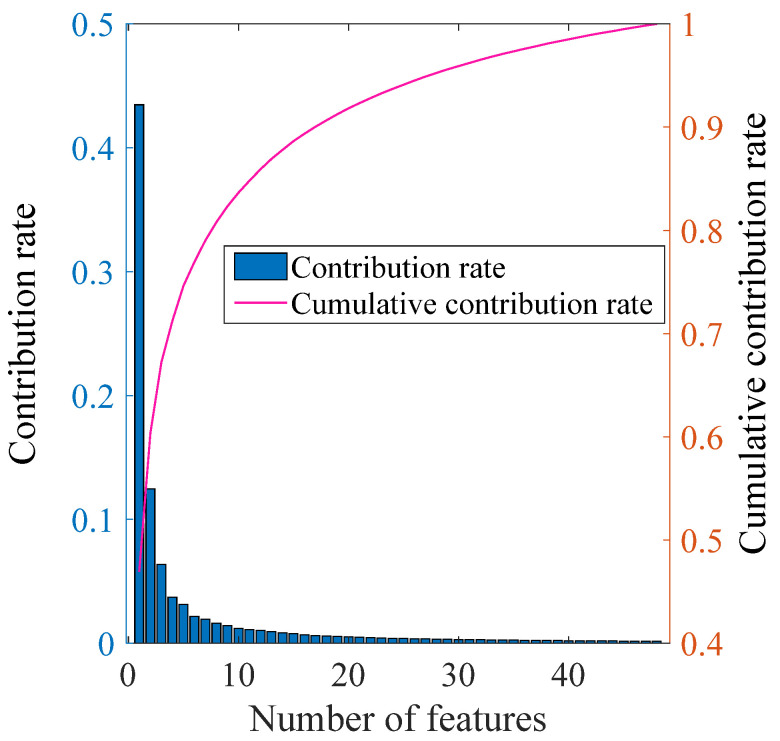
Feature distribution comparison pre- and post-PCA reduction.

After multilayer wavelet transform, a total of 5500 features were extracted. To address the potential issue of overfitting resulting from high dimensionality, the Principal Component Analysis (PCA) algorithm is applied for feature dimensionality reduction. Specifically, features with a cumulative contribution rate exceeding 98% are retained. Through this process, the original 1 × 5500-dimensional feature vector is compressed into a more manageable 1 × 50-dimensional feature vector, as depicted in Figure 5.

**Figure 6:**
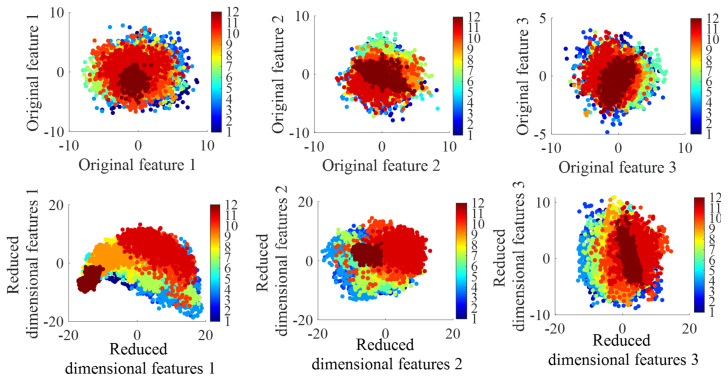
Dimensionality reduction graph based on PCA.

As illustrated in Figure 6, a visual comparison of the feature space distributions before and after dimensionality reduction is presented. The original features (prior to reduction) and the features reduced by PCA are visualized. The numerical labels ranging from 1 to 12 represent twelve different surface types: icy marble pavement, icy asphalt pavement, icy concrete, marble, rubber, asphalt, concrete, wet rubber, wet asphalt, wet concrete, gravel, and grassland. This graphical representation clearly demonstrates that the PCA-reduced features display significantly improved separability within the spatial domain when compared to the original features.

## 4. Feature-Level Fusion and Machine Learning Classifier

### 4.1. Dataset Construction

**Figure 7:**
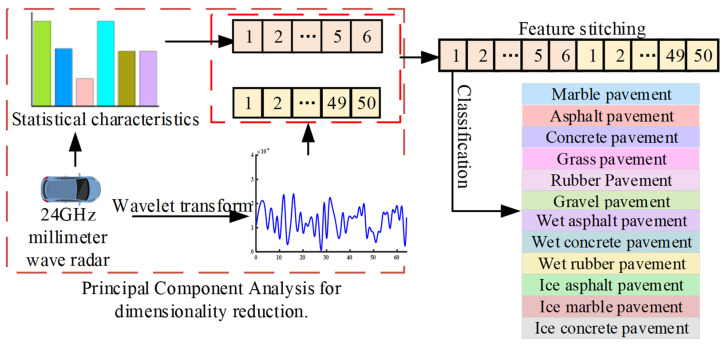
Feature-level fusion diagram of statistical features and wavelet-transformed features.

Through real-vehicle experiments, radar echo signals from various road types were gathered, yielding 8865 training samples. For each training sample, six-dimensional statistical features and fifty-dimensional wavelet-transformed features were extracted. Subsequently, these two types of features were combined to form a feature-level fusion vector, as shown in the 8865 × 56 matrix. Here, the number 8865 denotes the quantity of samples, and 56 represents the number of fused features, as shown in Figure 7.

### 4.2. Road Surface Recognition Classifier Training

To enhance the model’s robustness, the dataset is randomly partitioned, with 80% allocated for training and 20% designated for testing. Since the echoes of consecutive frames on the same driving trajectory have a strong correlation, we first group the data into “road sections” according to the vehicle’s driving path before dividing and ensure that all samples in the same road section only appear in one of the training set or the test set to avoid overfitting caused by samples with similar time series appearing in both training and testing. After division, the training set is cross-validated, and the above-mentioned road section grouping principle is still maintained. Samples of the same road section are assigned to the same fold to ensure that each verification is truly “new road section” data; after completing cross-validation, the optimal model is finally evaluated on an independent test set. This method avoids selecting features based on the distribution of the test set, thereby ensuring the unbiased nature of model development.

The performance of the classifier is assessed by calculating the average classification accuracy across five folds of the cross-validation process, which in turn determines the objective function value. The classifiers for this training are four classifiers selected based on the matlab classification learner toolbox: WNN, KNN, SVM, and Kernel. Upon completion of the training phase, the selected feature subset is rigorously evaluated using the independent test dataset. This comprehensive evaluation strategy guarantees the reliability and generalizability of the developed model.

**Figure 8:**
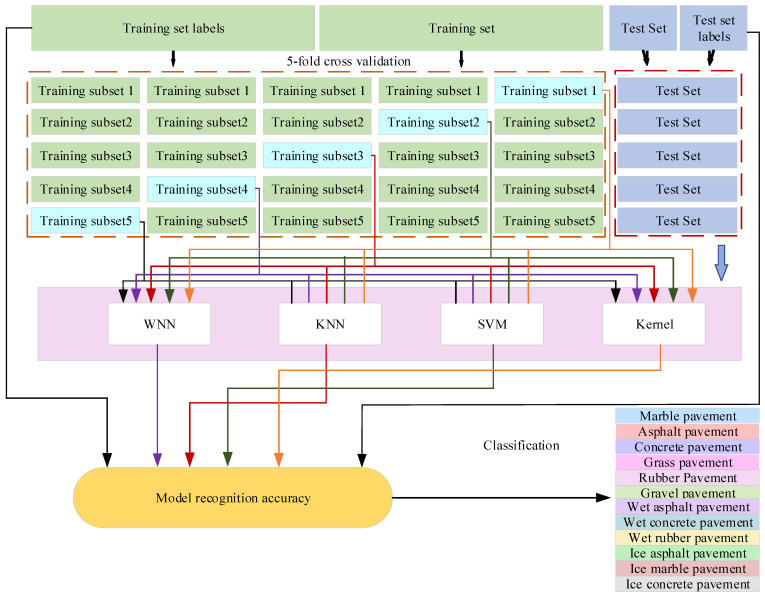
Five-fold cross-validation method for classification.

In the training process, a random five-fold cross-validation approach is adopted. To avoid overfitting, the training set is randomly partitioned into five subsets of equal size. Specifically, four of these subsets are utilized for the training phase, while the remaining subset is used for model validation, as illustrated in Figure 8.

The five-fold cross-validation procedure for the classifier is described as follows:

Step 1: The pavement type sample set is partitioned into a training set $S_{train}$ and a test set $S_{test}$ at a ratio of 4:1. Subsequently, the training set undergoes five-fold cross-validation, being randomly divided into five subsets, denoted as $S_{1}$, $S_{2}$, $S_{3}$, $S_{4}$, and $S_{5}$. Each subset will be employed as the validation set exactly once, with the other four subsets functioning as the training sets during each iteration. Specifically, it represents that $S_{i}\left(i=1,2,3,4,5\right)$ is the validation subset and $S_{+i}=S_{train}-S_{i}$ is the training s ubset.

Step 2: In this research, the classifiers selected for identifying pavement types consist of the Wide Neural Network (WNN), K-Nearest Neighbors (KNN), Support Vector Machine (SVM), and Kernel models. Each model is trained using the training subsets, and its performance is evaluated on the validation subset. Subsequently, the validation results are output. Simultaneously, the model is tested on the original test set, and the classification outcomes are documented.

Step 3: For each of the four models, Steps 1 and 2 are iteratively executed to generate classification results. Subsequently, these results are aggregated and averaged to precisely ascertain the final classification performance, ensuring a comprehensive and robust evaluation of the model’s effectiveness.

Table 2 summarizes the classification performance comparison of four machine learning classification algorithms (WNN, KNN, SVM, and Kernel-based classifiers) on 12 different road surfaces. Empirical results demonstrate that each method achieves an overall accuracy exceeding 80%, thereby evidencing the robustness of the fused statistical and wavelet-domain feature set. In particular, recognition rates for ice-covered concrete, wet concrete, gravel, and grass surfaces uniformly surpass 90%, with grass pavement yielding the highest precision (above 98%) across all models. This exceptional separability likely reflects grass’s low radar cross section and heterogeneous texture, which generate echo signatures that are both stable and readily distinguishable from those of rigid materials. Furthermore, the marginal differences in accuracy among WNN-, KNN-, SVM-, and Kernel-based approaches indicate that feature representation—rather than classifier architecture complexity—constitutes the primary determinant of recognition efficacy. These experiments demonstrate the feasibility of radar-based classifiers for real-time road surface classification.

**Figure 9:**
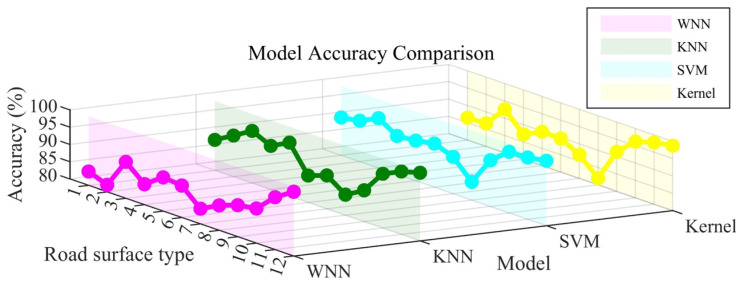
Training accuracy graph of different machine learning models.

The recognition accuracy of the four models is statistically analyzed and presented in a visual manner, as depicted in Figure 9. On the x-axis, the road identifiers are numbered from 1 to 12, each corresponding to a specific pavement type: 1 stands for Ice Marble, 2 for Ice Asphalt, 3 for Ice Concrete, 4 for Marble, 5 for Rubber, 6 for Asphalt, 7 for Concrete, 8 for Wet Rubber, 9 for Wet Asphalt, 10 for Wet Concrete, 11 for Gravel, and 12 for Grass. Meanwhile, the y-axis represents the model identifiers: 1 represents the WNN model, 2 the KNN model, 3 the SVM model, and 4 the Kernel model.

As illustrated in Figure 9, a comprehensive analysis reveals that all four models—WNN, KNN, SVM, and Kernel—achieve a recognition accuracy exceeding 80%. Among them, the SVM model, represented in blue, stands out prominently. It demonstrates remarkable consistency, delivering the highest accuracy across all pavement types. In most cases, its accuracy surpasses 90%, which underscores its robustness and exceptional performance under diverse pavement conditions.

The Kernel model, depicted in yellow, also exhibits a relatively high level of accuracy, although it slightly trails behind the SVM model. This indicates that it remains a reliable option for pavement type recognition. The KNN model, visualized in green, shows some degree of variability in accuracy when applied to different pavement types. Nevertheless, it generally maintains a commendable performance level, suggesting its adaptability to various scenarios.

Conversely, the WNN model, colored in purple, registers the lowest accuracy among the four models. Notably, it experiences substantial performance fluctuations for specific pavement types. These fluctuations imply that the WNN model may face challenges in accurately recognizing certain pavement categories, highlighting areas where its performance could potentially be improved.

**Figure 10:**
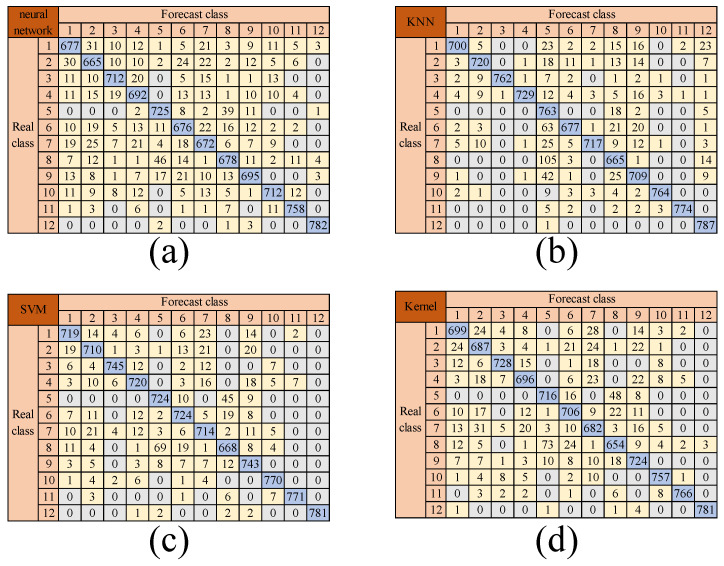
Confusion matrix plots for different validation models.

To conduct a more in-depth assessment of the models’ classification performance across various pavement types, the confusion matrices for model training are presented in Figure 10. On the confusion matrix, both the x-axis and the y-axis are marked with road identifiers ranging from 1 to 12. These identifiers correspond precisely to the same pavement types as previously described.

In the confusion matrix, the elements on the diagonal represent the samples that have been accurately classified, which is a clear indication of the model’s correct identification of the pavement types. In contrast, the off-diagonal elements signify the samples that have been misclassified. This distinction allows for a detailed and intuitive understanding of the models’ strengths and weaknesses in classifying different pavement types.

The quantitative analysis of the confusion matrix shows that all models achieved high accuracy in most categories. The wide neural network model correctly identified category 1 (ice marble pavement) 677 times, and the main diagonal elements of the confusion matrix were generally large, indicating that the model has strong classification ability in most categories. The KNN and SVM models also showed good recognition performance in the main categories, while the Kernel method maintained a relatively balanced distribution in the discrimination between categories. The analysis results also revealed that there was a certain degree of confusion between some adjacent categories in each model. The wide neural network model had 23 and 22 cross-classifications between category 3 (ice concrete) and category 4 (marble), respectively, and the KNN model had 39 misclassifications between category 5 (rubber) and category 6 (asphalt). This confusion may be due to the similarity in the characteristics of these pavement types. Ice-covered concrete and polished marble have highly similar diffuse reflectance characteristics in the visible spectrum, and dry rubber and asphalt also have the same dark gray tone and absorption peak distribution, which puts higher requirements on the model’s ability to distinguish between these types.

In summary, these high accuracy rates strongly demonstrate that all four models are very effective in distinguishing different road surface types, with slight deviations when distinguishing between classes with overlapping features, but are also able to reliably distinguish between slippery and icy roads, making them valuable tools for applications that require accurate road surface type identification.

## 5. Real Vehicle Test Verification

**Figure 11:**
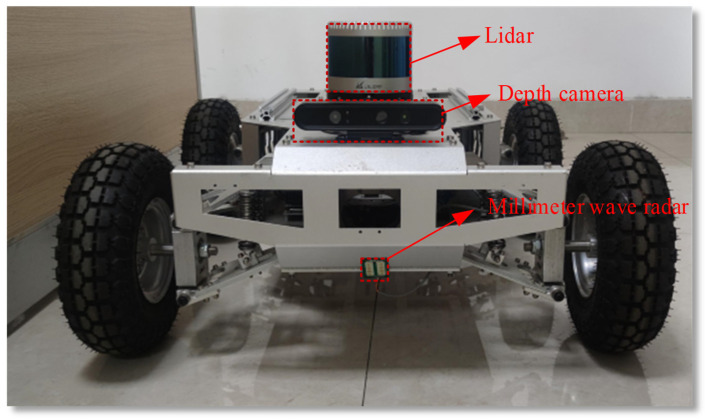
Real vehicle test platform diagram.

The experimental platform employed in this study is a remote-controlled chassis vehicle, as illustrated in Figure 11. This sophisticated platform is outfitted with an array of advanced hardware components. It features a 24 Hz millimeter-wave radar, which enables precise detection of objects in the surrounding environment. Complementing the radar is a Lidar system, capable of generating detailed 3D maps of the terrain. High-resolution cameras are also integrated, providing visual data for enhanced perception.

Moreover, the platform incorporates an inertial navigation system (INS)–GPS combined positioning system, ensuring accurate determination of its position, orientation, and movement in real time. An industrial computer serves as the central processing unit, handling the vast amounts of data collected by these sensors and facilitating seamless operation of the entire system. Together, these components form a comprehensive and highly capable experimental setup designed to support a wide range of research and testing activities.

In this study, the millimeter-wave radar takes center stage as the primary sensor for road surface detection. This sensor comes with a plethora of advantages. It is compact and lightweight, making it easy to integrate into the experimental setup. It boasts high resolution, enabling it to distinguish fine details of the road surface. The wide operating frequency band allows for a broad range of detection capabilities, while the large Doppler frequency response enhances its ability to detect moving objects or changes on the road. The short wavelength of the millimeter-wave radar is particularly beneficial as it can capture intricate target features, resulting in clear contour imaging of the road surface.

Based on empirical analysis, this study strategically installed the millimeter-wave radar at the front of the vehicle chassis to balance vibration suppression and echo quality. Specifically, when the installation height is less than 10 cm, chassis vibration is likely to cause echo noise to increase; when it exceeds 30 cm, the signal propagation path is extended, the attenuation is aggravated, and weak echoes are difficult to detect reliably. At the same time, a pitch angle that is too large may miss the echo of low-lying or slightly protruding objects, and a pitch angle that is too small is likely to produce a scattering blind area. Based on the above empirical considerations, this study ultimately determined the radar installation height to be 15 cm from the ground and the pitch angle to be 45°, in order to achieve comprehensive and accurate detection of the road ahead. The key parameters of the millimeter-wave radar are shown in Table 3.

To conduct a comprehensive assessment of the overall performance of the road surface recognition method proposed in this study, real campus road condition data were collected via the experimental vehicle platform, with the vehicle speed consistently maintained at an average of approximately 4 m/s. Four well-trained models were mainly subjected to testing: WNN, KNN, SVM, and Kernel methods. During the analysis, precision was adopted as the evaluation metric to quantitatively evaluate the performance of road surface type recognition.

**Figure 12:**
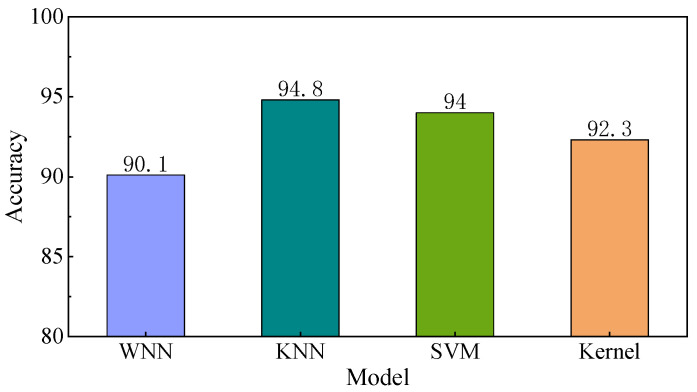
Comparison of typical model validation accuracy.

As depicted in Figure 12, the KNN model demonstrated the highest recognition accuracy, reaching 94.8%. This was closely followed by the SVM and Kernel models, which achieved accuracy of 94% and 92.3%, respectively. In contrast, the Wide Neural Network model attained a recognition accuracy of 90.1%. Remarkably, the test outcomes for all four models aligned well with the training results, yielding an average recognition accuracy of 92.8%. The absence of both overfitting and underfitting phenomena strongly suggests that the model training process was highly successful, validating the reliability and effectiveness of the training approach employed.

**Figure 13:**
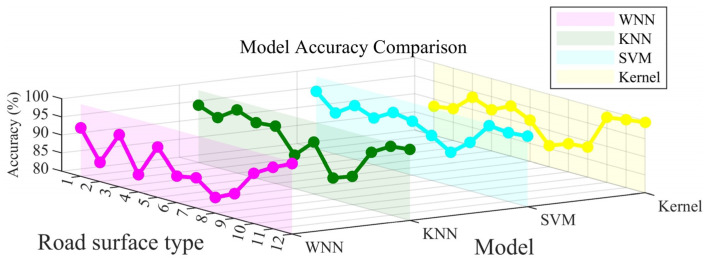
Comparison of validation accuracy of four models.

Figure 13 provides a comparative summary of the recognition accuracies of the four models across different road surface types. Each curve in the figure represents the recognition performance of a particular model. The WNN model (pink curve) shows relatively low and unstable accuracy, with noticeable fluctuations across different surface types. It does not exhibit a consistent advantage in any specific category. The Kernel (yellow) and KNN (green) models demonstrate generally similar overall performance, though their accuracies vary across different surface types. In particular, KNN achieves the highest mean accuracy but presents limited recognition capability for certain road surfaces, suggesting its strength lies more in overall generalization than in handling specific surface categories. The SVM model (blue) achieves consistently high recognition accuracy across most road surface types. While its margin over the other models may not always be large, it demonstrates relatively stable and reliable performance, indicating its potential as a robust choice for road surface recognition under the current feature setting.

**Figure 14:**
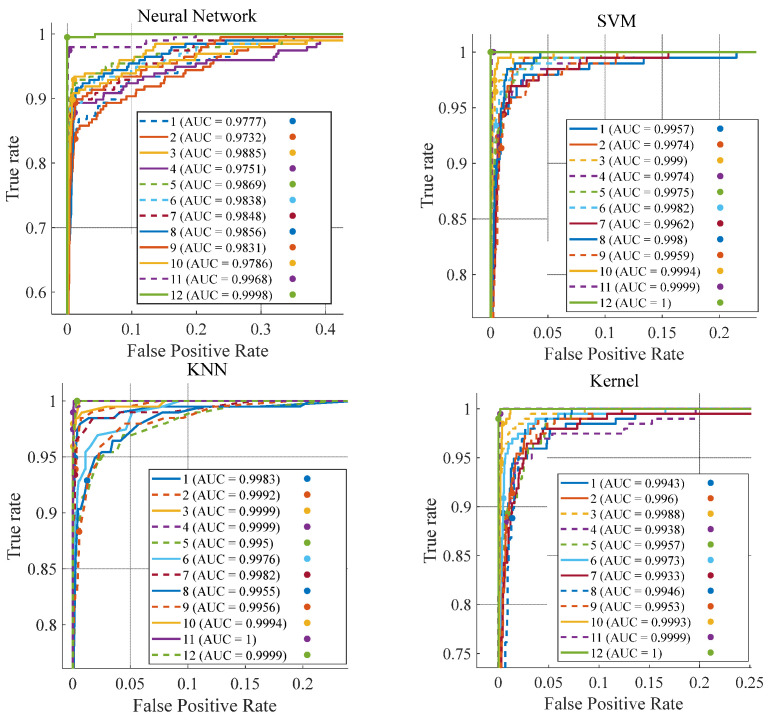
ROC curves of four models.

Furthermore, the Receiver Operating Characteristic (ROC) curves of the four models across diverse road surfaces were meticulously analyzed, as shown in Figure 14. These curves depict the trade-off between the True Positive Rate (TPR) and the False Positive Rate (FPR) of binary classification models at varying threshold values. The TPR, defined as the proportion of correctly classified positive samples among all positive samples, and the FPR, representing the proportion of negative samples misclassified as positive among all negative samples, are crucial metrics in evaluating the performance of these models.

In the ROC curves, the x-axis is designated to represent the FPR, while the y-axis denotes the TPR. Conceptually, an ideal classification model would have an ROC curve that closely approaches the upper-left corner of the plot, specifically the point (0, 1), as this position signifies the optimal scenario where the TPR is maximized and the FPR is minimized. The Area Under the Curve (AUC), a key performance metric for classification models, ranges from 0 to 1. A value approaching 1 indicates superior model performance, reflecting its effectiveness in correctly distinguishing between classes.

The numbered labels from 1 to 12 correspond to twelve distinct road surface types: ice marble, ice asphalt, ice concrete, marble, rubber, asphalt, concrete, wet rubber, wet asphalt, wet concrete, gravel, and grass. The classification performance of the four models was comprehensively evaluated across these twelve road surface categories.

The WNN model exhibits relatively modest AUC values. Among the tested road surfaces, it achieved its highest AUC of 0.9998 for grass and its lowest value of 0.9751 for marble. The WNN model lags behind the KNN and Kernel models in terms of performance.

The KNN model stands out with AUC values nearly reaching 1 for the majority of road surfaces, which is a strong indication of its exceptionally high classification accuracy. The Kernel model also demonstrates predominantly high AUC values, although its overall performance is marginally lower than that of the KNN model. Similarly, the SVM model shows high AUC values, albeit slightly less than those of the KNN model.

Overall, the ROC curves of all four models are positioned close to the upper-left corner of the plot, and their corresponding AUC values are relatively high. These results collectively underscore the models’ robust capabilities in effectively differentiating between various road surface types and conditions.

## 6. Conclusions

This paper introduces a multi-feature fusion method for road surface identification leveraging a 24 GHz millimeter-wave radar. By integrating statistical features with wavelet transform techniques, the method enables high-precision classification of 12 typical road surface types and conditions, achieving an overall identification accuracy of 94.2%. The proposed approach presents several key innovations.

Firstly, through in-depth statistical analysis, this study verifies the separability of millimeter-wave radar echo signals for different road surface types, facilitating the extraction of diverse statistical features. This foundational analysis provides a robust basis for subsequent feature engineering.

Secondly, a novel feature extraction strategy is proposed, which combines radar data reconstruction with wavelet transform. This innovative approach effectively enhances the spatio-temporal correlation of the signals, overcoming the limitations of traditional methods that often struggle to capture the fine-grained characteristics of road surfaces. As a result, it offers a more comprehensive representation of the road surface information.

Finally, the method capitalizes on the synergy of feature-level fusion and multiple machine learning models. This combination significantly improves both classification accuracy and robustness, enabling more reliable identification under various conditions.

Real-vehicle experimental results further validate the effectiveness of the proposed method. It demonstrates the ability to achieve efficient road surface identification using low-cost hardware setups. Among the evaluated models, the KNN model stands out, achieving the highest test accuracy of 94.8%. This result underscores the method’s superior performance, particularly in complex environments where reliable road surface identification is crucial.

The significance of this research lies in its development of a cost-effective road perception solution for intelligent vehicles and mobile robots. By enhancing the safety and reliability of autonomous driving systems, this solution not only fortifies the operational integrity of these platforms but also serves as a cornerstone for optimizing vehicle control strategies and elevating ride comfort.

In contrast to traditional visual sensors and Lidar, millimeter-wave radar exhibits remarkable resilience to environmental interference while maintaining a lower cost profile. This unique combination of attributes renders it especially well-suited for road monitoring in challenging weather conditions, including rain, snow, and fog. These capabilities expand the operational envelope of autonomous systems, ensuring reliable performance in environments where conventional sensors may falter.

In addition, a recent study proposed a hybrid method combining ultrasonic detection with artificial intelligence algorithms, using ultrasonic sensors to collect echo features in concrete-filled steel tubes (CFST) and predicting void defects through a Bayesian-optimized enhanced XGBoost model [42]. This method has achieved good results in building material integrity assessment. Its technical ideas are similar to the multi-scale microwave echo feature fusion strategy in this study, but there are differences in detection objects and sensor types. In the future, it can be considered to combine this method with the millimeter-wave radar feature fusion strategy of this study to expand the application of road surface and structural defect detection to a wider range.

Looking ahead, this method has great potential for development. Subsequent studies can further apply it to larger and more diverse road datasets and integrate other key road condition information (such as snow depth, pothole severity, etc.); at the same time, with the ability of deep learning in time-series modeling and feature expression, it is expected to further optimize the feature extraction algorithm and improve classification performance, thereby enhancing the versatility and accuracy of this method in complex environments.

## Figures and Tables

**Table 1:** Comparison of statistical characteristics of echo signals from different roads.

Road Surface Type	Average Value	Standard Deviation	Range Value	Median	Quartiles	Seventh Quartile
Concrete	8020.94	5872.46	22,421.27	6606.87	3124.5	12,216.23
Marble	9403.71	7466.94	29,010.04	7321.58	3483.61	13,524
Asphalt	8031.41	5146.02	22,615.45	6933.04	4176.95	10,811.43
Grass	3273.85	1622.93	7494.25	2987.36	2046.17	4215.78
Rubber	6634.26	2890.66	13,612.79	6347.72	4543.9	8465.43
Gravel	13,499.05	5793.34	38,027	12,686	9247	16,850.25
Wet asphalt	6992.37	4368.66	21,527	5978	3695.25	9702.75
Wet concrete	12,057.94	7075.07	30,550	11,537	5881.75	18,081.5
Wet rubber	7397.6	4287.82	27,568	6623	4294.75	9919.5
Ice marble	6623.7	5910.18	29,734	4719	1857.75	9635.25
Ice asphalt	8163.09	6072.19	27,927	6609	3489.75	11,620.75
Ice concrete	9677.6	8224.39	33,982	7018	2579.75	15,509.25

**Table 2:** Training accuracy of different models.

Road Surface Type	WNN (%)	KNN (%)	SVM (%)	Kernel (%)
Ice marble	84	88.8	90.9	86.5
Ice asphalt	82	91.9	91.8	86.7
Ice concrete	90.5	95.1	94.4	92.6
Marble	85.9	92.6	91.1	87.2
Rubber	89.7	95.4	91.6	89.8
Asphalt	89.2	87.7	92.6	89.7
Concrete	84.4	89.6	90.6	86.8
Wet rubber	87.2	85.9	85.2	81.9
Wet asphalt	89.1	89	93.3	91.4
Wet concrete	90	95.6	97.6	96.2
Gravel	95.1	98.1	97.8	97.8
Grass	98.5	99.6	98.7	98.7

**Table 3:** Main parameters of millimeter-wave radar.

Parameter	Numerical Value
Frequency (GHz)	24
Renewal rate (msec)	50
Actual detection distance (m)	<50
Transmit power (dBm)	10
Output voltage (V)	8–16

## Data Availability

Data will be available on request.

## Abbreviations

The following abbreviations are used in this manuscript:

WNN	Multidisciplinary Digital Publishing Institute
KNN	K-Nearest Neighbors
SVM	Support Vector Machine
CNN	Convolutional Neural Network
RBF	Radial Basis Function
BP	Backpropagation
MFCC	Mel-Frequency Cepstral Coefficient
ANN	Artificial Neural Network
CWT	Continuous Wavelet Transform
DWT	Discrete Wavelet Transform
PCA	Principal Component Analysis
ROC	Receiver Operating Characteristic
TPR	True Positive Rate
FPR	False Positive Rate
AUC	Area Under the Curve

## References

[1] Cao D., Wang X., Li L., Lv C., Na X., Xing Y., Li X., Li Y., Chen Y., Wang F.-Y. (2022). Future directions of intelligent vehicles: Potentials, possibilities, and perspectives. IEEE Trans. Intell. Veh..

[2] Ogunrinde I., Bernadin S. (2023). Deep camera–radar fusion with an attention framework for autonomous vehicle vision in foggy weather conditions. Sensors.

[3] Yu X., Salimpour S., Queralta J.P., Westerlund T. (2023). General-purpose deep learning detection and segmentation models for images from a LiDAR-based camera sensor. Sensors.

[4] Šabanovič E., Žuraulis V., Prentkovskis O., Skrickij V. (2020). Identification of road-surface type using deep neural networks for friction coefficient estimation. Sensors.

[5] Marsh B., Sadka A.H., Bahai H. (2022). A critical review of deep learning-based multi-sensor fusion techniques. Sensors.

[6] Obet R., Juliandy C., Ndi A., Endri H., Hendrik J., Tarigan F.A. (2022). Image road surface classification based on GLCM feature using LGBM classifier. IOP Conf. Ser. Earth Environ. Sci..

[7] Chen C., Seo H., Jun C.H., Zhao Y. (2022). Pavement crack detection and classification based on fusion feature of LBP and PCA with SVM. Int. J. Pavement Eng..

[8] Zagoruyko S., Komodakis N. Learning to compare image patches via convolutional neural networks. Proceedings of the 2015 IEEE Conference on Computer Vision and Pattern Recognition (CVPR).

[9] Gao L., Song W., Dai J., Chen Y. (2019). Road extraction from high-resolution remote sensing imagery using refined deep residual convolutional neural network. Remote Sens..

[10] Zhao J., Wu H., Chen L. (2017). Road surface state recognition based on SVM optimization and image segmentation processing. J. Adv. Transp..

[11] Liu X.Y., Huang Q.D. (2011). Study on classifier of wet-road images based on SVM. J. Transp. Sci. Eng..

[12] Wan J., Zhao K., Wang W.F. (2013). Classification of slippery road images based on high-dimensional features and RBF neural network. Transp. Inf. Saf..

[13] Wang X.W., Li S.Y., Liang X., Li S.H., Zheng J.J. (2024). A complex pavement rapid recognition model based on structural reparameterization and adaptive attention. China J. Highway Transp..

[14] Aki M., Rojanaarpa T., Nakano K., Suda Y., Takasuka N., Isogai T., Kawai T. (2016). Road surface recognition using laser radar for automatic platooning. IEEE Trans. Intell. Transp. Syst..

[15] Liu B., Zhao D., Zhang H. (2023). Road classification using 3D LiDAR sensor on vehicle. Meas. Sci. Technol..

[16] Lalonde J.F., Vandapel N., Huber D.F., Hebert M. (2006). Natural terrain classification using three-dimensional ladar data for ground robot mobility. J. Field Robot..

[17] Stove A.G. (1992). Linear FMCW radar techniques. IEE Proc. F Radar Signal Process..

[18] Kim M.H., Park J., Choi S. (2021). Road type identification ahead of the tire using D-CNN and reflected ultrasonic signals. Int. J. Automot. Technol..

[19] Schneider R., Didascalou D., Wiesbeck W. (2000). Impact of road surfaces on millimeter-wave propagation. IEEE Trans. Veh. Technol..

[20] Harikrishnan P.M., Gopi V.P. (2017). Vehicle vibration signal processing for road surface monitoring. IEEE Sens. J..

[21] Han K., Choi M., Choi S.B. (2018). Estimation of the tire cornering stiffness as a road surface classification indicator using understeering characteristics. IEEE Trans. Veh. Technol..

[22] Gueta L.B., Sato A. Classifying road surface conditions using vibration signals. Proceedings of the 2017 Asia-Pacific Signal and Information Processing Association Annual Summit and Conference (APSIPA ASC).

[23] Ao X., Wang L.M., Hou J.X., Xue Y.Q., Rao S.J., Zhou Z.Y., Jia F.X., Zhang Z.Y., Li L.M. (2023). Road recognition and stability control for unmanned ground vehicles on complex terrain. IEEE Access.

[24] Wang B., Guan H., Lu P., Zhang A. (2014). Road surface condition identification approach based on road characteristic value. J. Terramech..

[25] Gailius D., Jacenas S. (2007). Ice detection on a road by analyzing tire to road friction ultrasonic noise. Ultragarsas.

[26] Alonso J., López J.M., Pavón I., Recuero M., Asensio C., Arcas G., Bravo A. (2014). On-board wet road surface identification using tyre/road noise and support vector machines. Appl. Acoust..

[27] Zhang L., Guan K.R., Lin X.L., Guo P.Y., Wang Z.P., Sun F.C. (2023). Method for estimating pavement adhesion coefficient based on the fusion of image recognition and dynamics. Automot. Eng..

[28] Safont G., Salazar A., Rodriguez A., Vergara L. A multisensor system for road surface identification. Proceedings of the 2019 International Conference on Computational Science and Computational Intelligence (CSCI).

[29] Yanik M., Chandrasekaran A.K., Rao S. (2023). Machine Learning on the Edge with the mmWave Radar Device IWRL6432.

[30] Jaén-Vargas M., Leiva K.M.R., Fernandes F., Gonçalves S.B., Silva M.T., Lopes D.S., Olmedo J.J.S. (2022). Effects of sliding window variation in the performance of acceleration-based human activity recognition using deep learning models. PeerJ Comput. Sci..

[31] Guo T., Zhang T., Lim E., López-Benítez M., Ma F., Yu L. (2022). A review of wavelet analysis and its applications: Challenges and opportunities. IEEE Access.

[32] Huang J., Jiang B., Xu C., Wang N. (2021). Slipping detection of electric locomotive based on empirical wavelet transform, fuzzy entropy algorithm and support vector machine. IEEE Trans. Veh. Technol..

[33] Panigrahi B.K., Ray P.K., Rout P.K., Mohanty A., Pal K. (2018). Detection and classification of faults in a microgrid using wavelet neural network. J. Inf. Optim. Sci..

[34] Padhy S.K., Panigrahi B.K., Ray P.K., Satpathy A.K., Nanda R.P., Nayak A. Classification of faults in a transmission line using artificial neural network. Proceedings of the 2018 International Conference on Information Technology (ICIT).

[35] Osadchiy A., Kamenev A., Saharov V., Chernyi S. (2021). Signal processing algorithm based on discrete wavelet transform. Designs.

[36] Daubechies I. (1992). Ten Lectures on Wavelets.

[37] Mallat S. (1999). A Wavelet Tour of Signal Processing.

[38] Martinez-Ríos E.A., Bustamante-Bello R., Navarro-Tuch S., Perez-Meana H. (2022). Applications of the Generalized Morse Wavelets: A Review. IEEE Access.

[39] Raath K.C., Ensor K.B. (2020). Time-Varying Wavelet-Based Applications for Evaluating the Water-Energy Nexus. Front. Energy Res..

[40] Mallat S.G. (2002). A Theory for Multiresolution Signal Decomposition: The Wavelet Representation. IEEE Trans. Pattern Anal. Mach. Intell..

[41] Sang Y.F., Singh V.P., Sun F., Chen Y., Liu Y., Yang M. (2016). Wavelet-Based Hydrological Time Series Forecasting. J. Hydrol. Eng..

[42] Wan S., Li S., Chen Z., Tang Y., Wu X. (2025). An Ultrasonic-AI Hybrid Approach for Predicting Void Defects in Concrete-Filled Steel Tubes via Enhanced XGBoost with Bayesian Optimization. Case Stud. Constr. Mater..

